# New oral anticoagulants: discussion on monitoring and adherence should start now!

**DOI:** 10.1186/1477-9560-11-8

**Published:** 2013-06-28

**Authors:** Hugo ten Cate

**Affiliations:** 1 Department of Internal Medicine, Laboratory for Clinical Haemostasis and Thrombosis, Maastricht University Medical Center, PO Box 616, Maastricht, MD, 6200, the Netherlands; 2 Cardiovascular Research Institute Maastricht, PO Box 616, Maastricht, MD, 6200, the Netherlands

**Keywords:** New oral anticoagulants, Adherence, Vitamin K antagonists, Laboratory assays

## Abstract

New oral anticoagulants (NOACs) have been introduced to improve anticoagulant therapy worldwide, but safe implementation may require additional measures. First, optimization of dose adjustment based on therapeutic levels of the drug may be more appropriate than fixed dose therapy. The development and implementation in quantitative laboratory assays will enable further dose optimization. Second, non-adherence to medication is a potential threat to the safe use of NOACs. Since cardiovascular medication may not be optimally used in about 50% of patients, procedures to improve adherence are imperative, also for NOAC therapy and in particular in elderly patients.

## Background

Since mid 20^th^ century, vitamin K antagonists (VKA) have been introduced to become the main type of anticoagulant therapy for the prevention and treatment of thrombotic disorders [1]. From onset, VKA therapy has been monitored by means of a prothrombin clotting time, aiming for a certain degree of prolongation. Given interindividual variation in PT responses, individual dose adjustment of VKA was warranted. This prothrombin time based laboratory monitoring further evolved towards a more standardized way of testing, based on the International Normalized Ratio (INR). Over the past decades, INR adjusted VKA therapy has remained the exclusive form of oral anticoagulant therapy [2,3]. Obviously, the requirement of INR dose adjustment was felt as a practical burden to both patient and physician. In particular, in areas of the world where proper INR management is difficult or even impossible to achieve, the idea of fixed dose anticoagulation is attractive. Also for this reason, studies were undertaken addressing the potential value of fixed dose VKA administration. Unfortunately, although some of these studies suggested a potential benefit, the ultimate verdict is negative. Fixed dose warfarin failed to demonstrate a clinically significant antithrombotic effect for diverse indications including atrial fibrillation, myocardial infarction and catheter related thrombosis [4-10].

New oral anticoagulants (NOACs) have been developed in order to improve medical treatment of patients at risk of (recurrence) thrombotic disorders. One of the principles applied in the clinical testing of NOACs has been the application of a fixed dose, with some possibility for dose adjustment based on clinical criteria (renal function and/or specific interactions) [11]. Subsequent large scale clinical trials established the efficacy and safety of fixed doses of dabigatran, rivaroxaban and apixaban, for preventing ischemic stroke [12-14] and (recurrent) venous thromboembolism [15-18] as compared to INR titrated warfarin. This opened the way for registration of these drugs in the chosen doses for these indications. Currently, in many countries dabigatran, rivaroxaban and apixaban are registered for AF and/or prevention and/or treatment of venous thromboembolism, while studies with edoxaban are nearing completion. NOACs are being prescribed in many countries, in part replacing VKA, although a considerable number of indications for VKA remain, largely determined by indications not studied for NOAC’s, and/or by exclusion criteria applied in the phase 3 trials.

### Fixed dose: ideal dose?

From a theoretical point of view a fixed dose policy is remarkable for the class of anticoagulants. In fact, there are no convincing arguments in favor. In contrast to most platelet inhibitors, the effect of the NOAC’s is not irreversible, so there is no all or nothing effect. Kinetically, one would expect a dose response effect, so that an optimal drug level should be obtained to obtain the correct balance between antithrombotic efficacy and bleeding risk (like with VKA). Also, the poor experience with fixed doses of VKA should not have encouraged efforts to prove that fixed doses of anticoagulants are ideal.

Several arguments in favor of fixed dosing may also be given. Overall, indeed, a more predictable and perhaps more stable pharmacokinetic (PK) profile may be expected from NOACs as compared to VKA, for various reasons including lack of food interactions and fewer medication related interactions. Finally, the clinical trials with NOACs essentially prove the safety of the fixed dose policy; however, the question may be valid whether this safety (in practice) could be further improved taking into account individual PK?

In PK studies with dabigatran [19-24], rivaroxaban [25-28] and apixaban [29-31] marked interindividual variation in drug levels has been observed in healthy persons, as well as in subjects with liver or renal disease.

PK data obtained in the large clinical trials also show considerable interindividual variation in drug levels and activities (measured by clotting tests and/or aXa assay). Published data show the range in responses to dabigatran for the indication stroke prevention (150 mg bd), with average peak and trough levels of 175 and 91 ng/ml (assayed by TT-Hemoclottest), with 25-75^th^ percentile (ng/ml) of 117–275 and 61–143 ng/mL, respectively [32,33]. Mean levels of dabigatran were much higher in those with major bleeding versus those without bleeding complications (trough 141 ng/mL, SD 97.7 and 92.4, SD 67.9, respectively, RE-LY data total dabigatran groups).

For rivaroxaban, concentration levels determined by HPLC on clinical samples have now been released, not for stroke yet. For VTE treatment and prevention of recurrent VTE at a dose of 20 mg once daily, the average concentration is 215 ug/L peak (90% predicted interval 22–535 ug/L) and a trough of 32 (6–239 ug/L) [34].

Given these data in relation to clinical trial outcome, are the tested NOAC doses also the ideal doses? This is an important question as due to the more predictable and postulated stable PK, it may be foreseen that patients that on average respond within the preferred concentration range (which has not been defined for the different compounds) will be well protected at an acceptable risk of recurrent thromboembolism and bleeding, respectively. The argument here is that the therapeutic window is wider than for VKA. However, given the marked variation between individuals one must assume that a significant fraction of patients will be exposed to either too low or too high drug levels. Given the postulated stability in PK, this means that such subjects will be consistently exposed to suboptimal drug levels for the duration of their treatment. This also implicates that improvement in drug management must be considered, based on individual drug level determination.

From a registration point of view this is a difficult dilemma, as not the drugs, but *specific doses* of NOACs, were approved by FDA and EMA. As an example, the FDA only approved the 150 mg dabigatran dose for AF stroke prevention [35], whereas in Europe both the 110 and 150 mg doses were approved.

The available recommendations for NOAC dose adjustment in specific situations like deteriorating renal function or interactions (e.g. amiodarone) do not solve the more fundamental question as to why we would not try to give the optimal dose of a drug to the individual patient in the first place, potentially further improving the clinical efficacy risk profile. The fact that warfarin needs dose adjustment by INR does not disqualify this agent in itself; it is a matter of inconvenience, but trying to optimize drug levels within individual patients remains a useful aim. The advantage for NOAC’s may be that after initial dose optimization based on drug levels, this may be maintained without further adjustments for a prolonged period, assuming renal function stability.

### Where to go?

#### Follow up and testing

The clinical community is still trying hard to manage the NOACs by developing protocols for dose adjustment, indications for testing, dealing with bleeding episodes and so. Forced by publicity and pharmaceutical pressure, clinicians and patients need to figure out how to proceed in practice with drugs that do not require any monitoring, except for an occasional check on adherence by the pharmacist and a regular renal function check (although this may not be the ideal management). In our country the entire follow up of patients on NOACs (reimbursed only for AF at this stage) is put in the hands of community pharmacists and general practitioners (and/or nursing home specialists) [36]. Since cardiologists will not keep the majority of AF patients under regular surveillance, unless there are complicating factors, the patient may lack proper surveillance with regard to side effects, complications, adherence etcetera. This situation is disturbing given the fact that long term medication is prone not to be used properly by ± 50% of the patients! [37-39] While there are many ways by which adherence to medication can be beneficially influenced this has not yet been an issue of general concern with the NOACs.

Knowing the facts however about non-adherence for cardiovascular medication (and why would this be different for anticoagulation?), what can be done to optimize the situation? Of course, the absence of laboratory test burden is a relief for the doctor and patient alike. However, current recommendations of renal control 2–3 times/year already indicate that one cannot fare well without any testing [36]. In addition, as stated by some authors there are many conditions where temporary deterioration in renal function may occur in the elderly, for instance during intercurrent illnesses with diarrhea [40]. Here, the lessons from VKA therapy should warn us that any form of comorbidity may have serious consequences for drug intake, absorption and metabolism, in general, certainly in the elderly.

Current recommendations for laboratory testing aimed at measuring drug levels or the anticoagulant responses of NOAC’s are focused on the patients that either undergo interventions and/or have bleeding complications. At the same time the list of potential indications for testing can be easily expanded [40], such that in the average 75+ patient, 5–10 lab tests per year may be warranted. Hence, the advantage of being “unmonitored” while on NOAC’s may only be correct for the relative healthy, young patient with AF (or VTE), but it is prone to fail in the elderly.

Meanwhile, many laboratories throughout the world are actively arranging laboratory assays to become available. These comprise routine assays like aPTT and PT, provided reagents are sensitive to detect the effects of NOAC’s [41,42]. For quantitative purposes several assays are commercially available, including a modified thrombin clotting time for dabigatran and anti-Xa based assays for FXa inhibitors. Using specific calibrators therapeutic levels of NOACs can thus be measured. While point of care assays are particularly wanted for emergency settings, quantitative assays may find a place for eventual dose adjustment purposes. The idea of implementing laboratory testing for NOACs on a routine basis is currently not useful, in the absence of therapeutic target levels (and because specific dose regimens instead of variable doses, have been registered).

#### Adherence to NOAC’s?

Amazingly little literature is available regarding adherence to anticoagulant therapy. While there is an array of published reports and expert committee discussion papers on non-adherence of medication (as summarized in 37–39), particularly for the long term, there is no database on anticoagulants. Apparently, the general perception is that VKA monitoring with INR provides a tool for maintaining patients adherent. On the other hand, it is commonly known that adherence cannot be forced on persons by single measurements. A good example is the so-called white coat adherence of patients to antihypertensive drugs (summarized in the recent think-tank paper, 38). A time window of about 5 days before and after the blood pressure reading conveys a short-term adherence effect [38]. In analogy with blood pressure monitoring, more sustainable forms of adherence stimulation are required, also in current patients on VKA therapy.

Consensus is that with CV medication for chronic use the non-adherence rate adds up to 50%, translating to about 125.000 deaths in the USA annually [37,38]. The total costs of non-adherence range between 100 and 300 billion US$ per year. It has also been estimated that with every extra dollar spent on adherence enhancing measures an average of about 4–7 US$ is saved in preventing major complications and death in diseases including diabetes, hypertension and hypercholesterolemia [37,38]. Many individual factors determine adherence and overall, there are no major differences in these factors per type of indication or population. Motivation to take medication is for one determined by the perception of symptoms. In case of hypertension, patients are more motivated to continue medication if they perceive a certain benefit (reduced headache, less palpitations etc.). However, in the absence of such symptoms, adherence drops and this may occur with NOAC therapy, where (possibly with the exception of the acute phase of VTE), symptoms are absent (most apparently in AF patients). Thus, it can be expected that in the management of NOAC’s non-adherence may reach comparable figures (±50%) if no measures to boost adherence are being taken. The argument that in trials fixed dose therapy was as good as monitored warfarin does not take into account that trial patients are motivated, literate and get frequent follow up attention by trial nurses about any side effects etcetera. Moreover, potentially non-adherent patients were excluded, at least in some trials. Hence, these trial data are unlikely to predict real world adherence.

While NOAC’s are being prescribed worldwide it is uncertain whether accurate data on complications due to non-adherence will be recorded. Although major bleeding complications have been noted and debated publicly, the absence of the comparison with VKA users in real life makes sensible conclusions virtually impossible. This is even more so with thromboembolic complications. It is therefore highly unlikely that changes in incidence of stroke and/or bleeding will raise suspicion if occurring in patients that are at high risk for such complications to begin with. Thus, it does not make sense to wait for complications to raise awareness regarding the above issues.

What should be done? For a start, adherence should become a major topic of discussion, as it has been in the US. In every country where these drugs are licensed for use, policy makers, consumers, physicians and insurers should take their responsibility and start discussing the options for maximizing adherence, preferably in a patient centered manner. Solutions may not be simple, but this should not prevent searching for the best strategies.

## Concluding remarks

NOACs have been introduced to improve anticoagulant therapy worldwide. In particular in countries where current VKA control is difficult to organize, NOACs may provide a promising alternative. Two issues need to be taken into account in order to obtain safe anticoagulation with NOACs (or VKA). First, for NOACs therapeutic ranges of each agent should become available based on concentrations and/or dose response effects in laboratory tests. This will ultimately provide a means of optimizing dose adjustment in individual patients, more so than by current algorithms.

Secondly, stimulation of adherence is of utmost importance. Given the body of data showing poor adherence in patients on long term medication, similar problems may be expected in those using NOACs. This requires measures to prevent non-adherence, preferably in a patient centered manner. Further discussion and studies are needed to raise awareness for this adherence to medication problem, amongst patients, authorities and prescribers.
